# Quality Improvement in the Preoperative Evaluation: Accuracy of an Automated Clinical Decision Support System to Calculate CHA_2_DS_2_-VASc Scores

**DOI:** 10.3390/medicina58091269

**Published:** 2022-09-13

**Authors:** Chantal van Giersbergen, Hendrikus H. M. Korsten, Ashley. J. R. De Bie Dekker, Eveline H. J. Mestrom, R. Arthur Bouwman

**Affiliations:** 1 Department of Anesthesiology, Pain Medicine and Intensive Care, Catharina Hospital, 5623 EJ Eindhoven, The Netherlands; 2 Department of Anesthesiology, Maastricht University Hospital, 6229 HX Maastricht, The Netherlands; 3 Department of Signal Processing Systems and Electrical Engineering, Signal Processing Systems TU/e University of Technology, 5612 AZ Eindhoven, The Netherlands

**Keywords:** CHA_2_DS_2_-VASc, decision support system, quality, perioperative screening

## Abstract

*Background and Objectives*: Clinical decision support systems are advocated to improve the quality and efficiency in healthcare. However, before implementation, validation of these systems needs to be performed. In this evaluation we tested our hypothesis that a computerized clinical decision support system can calculate the CHA_2_DS_2_-VASc score just as well compared to manual calculation, or even better and more efficiently than manual calculation in patients with atrial rhythm disturbances. *Materials and Methods*: In *n* = 224 patents, we calculated the total CHA_2_DS_2_-VASc score manually and by an automated clinical decision support system. We compared the automated clinical decision support system with manually calculation by physicians. *Results*: The interclass correlation between the automated clinical decision support system and manual calculation showed was 0.859 (0.611 and 0.931 95%-CI). Bland-Altman plot and linear regression analysis shows us a bias of −0.79 with limit of agreement (95%-CI) between 1.37 and −2.95 of the mean between our 2 measurements. The Cohen’s kappa was 0.42. Retrospective analysis showed more human errors than algorithmic errors. Time it took to calculate the CHA_2_DS_2_-VASc score was 11 s per patient in the automated clinical decision support system compared to 48 s per patient with the physician. *Conclusions*: Our automated clinical decision support system is at least as good as manual calculation, may be more accurate and is more time efficient.

## 1. Introduction

Computerized clinical decision support systems (CCDSS) aim to support healthcare professionals in decision-making in clinical practice. As electronic health records (EHR) have largely replaced paper health records, sufficient reliable clinical data registries from multiple data sources are now available for CCDSS to support clinicians in their daily practice. In the present study, we analyze whether a CCDSS can be used for calculating risk scores such as in this case the CHA_2_DS_2_-VASc score.

Checklists are one of the oldest decision support tools. Over time checklists have evaluated from static paper lists in 1940’s to intelligent dynamic checklists [1,2]. The modality of a checklist serves as an ideal method for CCDSS to communicate the output of computer algorithms to advise clinicians in real-time at the bedside. Recently, we demonstrated that intelligent dynamic checklists in an intensive care (ICU) environment improved the adherence to best practice by increasing the percentage of checked items from 75% using traditional paper checklists to a 100% score [3,4]. Importantly, when the physicians used the dynamic intelligent checklist, there were no aspects overlooked during the daily ICU rounds.

In this manuscript, we used a CCDSS to automatically calculate the CHA_2_DS_2_-VASc score (further mentioned as automated clinical decision support system (aCDSS)). Just like an ICU round, there are a lot of items that needs to be checked during a preoperative evaluation. These checks cost time, manpower and are prone for human errors or omitted checks. The transition towards EHR facilitates the application of aCDSS that can perform the calculations of risk score more accurately and efficiently.

In this manuscript we compare a manual calculation of CHA_2_DS_2_-VASc scores (manual clinical decision support system (mCDSS)) in patients with atrial rhythm disturbances with an automatic calculation performed by a transparent intelligent algorithm from a aCDSS during a perioperative screening outpatient clinic visit. We hypothesize that the manual calculations contain more erroneous scores than aCDSS due to human errors, while in the meantime the automated calculation is also more efficient.

## 2. Materials and Methods

### 2.1. Patient Selection

Patients with atrial rhythm disturbances were included in this analysis when they visited the pre-operative outpatient clinics at the Catharina hospital Eindhoven from October 2017 until January 2018.

Written informed consent was given by all subjects.

Ethical committee approved this study nWMO-2017.81 (local) and W17.144 (national: MEC-U), received 30 November 2017.

#### 2.1.1. Automated CHA_2_DS_2_-VASc Score Calculation

**Figure 1:**
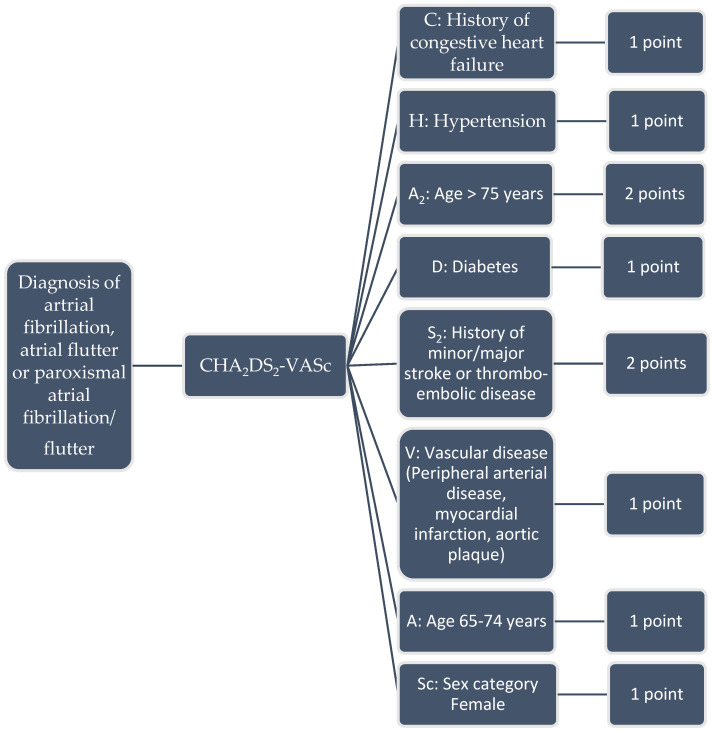
CHA_2_DS_2_-VASc score and scoring system (range 0–10).

**Figure 2:**
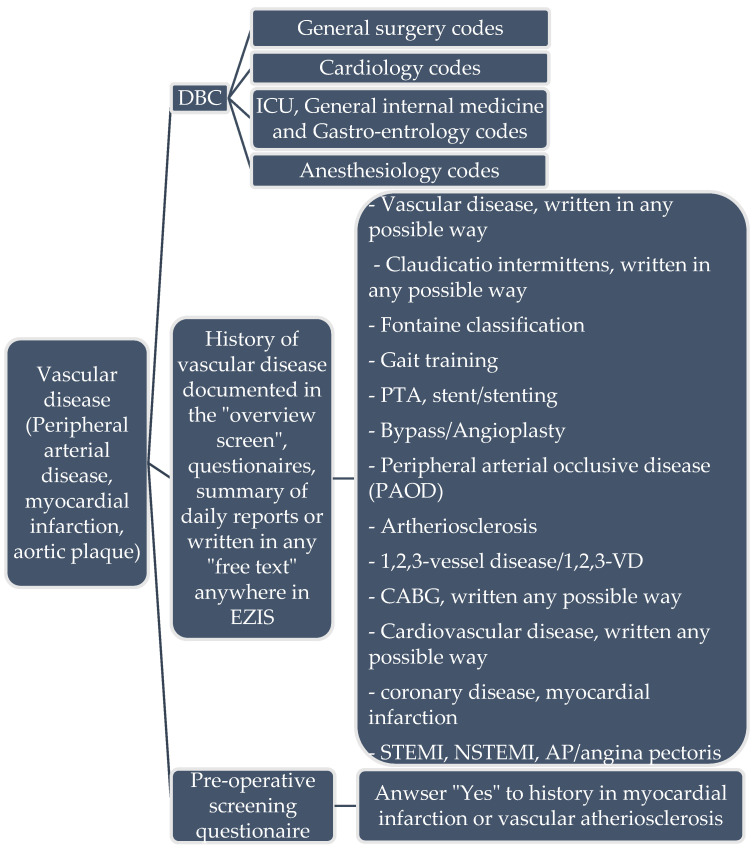
Example of information input to Gaston for extracting data from EZIS. DBC: Diagnose treatment combination (code for the healthcare insurance in the Netherlands to determine healthcare costs). PTA: percutaneous transluminal angioplasty.

Clinical rules to calculate the CHA_2_DS_2_-VASc were constructed using an aCDSS [5] designed in the Netherlands (by Gaston Medical, Eindhoven, The Netherlands). Gaston is an aCDSS that was applied in the Catharina hospital Eindhoven as a Pharmalogical support system to analyse errors in prescribing medication in EZIS (Elektronische Ziekenhuis Informatie Systemen; a EHR) created by Chipsoft Holding B.V., Amsterdam, The Netherlands) [3,6]. In case of an error, Gaston notifies pharmacists or even give pop-ups in the EHR to alert physicians. It can also screen the EHR and other medical data sources for targeted extraction and analysis of medical data to evaluate each of the CHA_2_DS_2_-VASc criteria. For this study we included data like the sex, age, hypertension (including measurement in vital parameters > three times systolic blood pressure above 140 mmHg and/or diastolic blood pressure > 90 mmHg), diabetes (including laboratory results fasting glucose > 6 mmol/L and/or normal glucose > 8 mmol/L), thromboembolic disorders (cerebrovascular accident, transient ischemic attack, long embolus and deep venous thrombosis), heart failure, symptomatic arteriosclerosis in the legs, aorta and symptomatic coronary disease (Figure 1). Besides structured data GASTON can extract information from free-text electronic hospital health record notes such as a medical condition or complications [7]. Gaston uses a rule engine with various clinical rules consisting of manually build transparent algorithms that are comparable with a decision tree (Figure 2). The whole system was designed to create or modify the rules easily. The rule engine can manually or automatically be activated to run the algorithm to screen, extract and analyze all relevant data [8]. Then Gaston calculates the CHA_2_DS_2_-VASc score and report the result in the EHR, as an item in an intelligent dynamic checklist, or alert the physician with a pop-up. A detailed description of this process for this study is described in Supplementary S1.

#### 2.1.2. Manual CHA_2_DS_2_-VASc Score Calculation

Three physicians were asked to manually calculate the CHA_2_DS_2_-VASc scores by evaluating the EHR and other available data sources. They reported the time they needed to analyze one single patient, each separate item of the CHA_2_DS_2_-VASc score, and the total CHA_2_DS_2_-VASc score.

### 2.2. Outcomes

The primary outcome of this study is to evaluate if the automatically calculated CHA_2_DS_2_-VASc score is as accurate as a manually calculated score which is the current medical standard of practice. Secondary outcome measurements are the time to calculate the CHA_2_DS_2_-VASc score automatically and manually, and a comparison between the CCDSS and the manual evaluation to determine the degree of correlation for the following CHA_2_DS_2_-VASc score criteria: hypertension, diabetes, thromboembolic disorders (cerebrovascular accident, transient ischemic attack, long embolus and deep venous thrombosis), heart failure, symptomatic arteriosclerosis in the legs and symptomatic coronary disease.

### 2.3. Statistical Analysis

For statistical analysis SPSS (IBM version 25) was used. Agreement between total CHA_2_DS_2_-VASc score of each patient, comparing our aCDSS with mCDSS, was assessed by calculating intraclass correlations. We categorized the total CHA_2_DS_2_-VASc score in three groups: 0 (no anticoagulants necessary), 1–7 (coagulants necessary, but no need to bridge when temporarily stopped) and >7 (coagulants necessary, but indication to bridge with LMWH (low molecular weight heparins) when temporarily stopped). A Bland-Altman plot and regression analysis were used to visualize the agreement between the two different interventions (aCDSS vs. mCDSS). The one-sample t tests from the Bland-Altman plot are shown in Table 1 and Table 2. The regression coefficients analysis was performed determine the level of agreement between the total CHA_2_DS_2_-VASc scores of both interventions. We also calculated the interclass correlation of our aCDSS and mCDSS regarding CHA_2_DS_2_-VASc score criteria. The investigators performed a comprehensible evaluation of the EHR by unblinding them to Gaston’s output to re-calculate the CHA_2_DS_2_-VASc score in the patients where the results of the calculations differed between the two interventions. These reviewed and re-calculated scores were then considered by mutual agreement between the investigators to be the true CHA_2_DS_2_-VASc score to clarify if the differences were due to a human or an algorithmic error.

## 3. Results

In total, 224 patients were selected for analysis. The mean age was 72, of whom 67% was male and the ASA (Physical Status Classification System) ranged from 2 to 4. The three physicians divided the 224 patients equally for their manual evaluations.

The interclass correlation of aCDSS and mCDSS of the total CHA_2_DS_2_-VASc score, was 0.859 (95%-CI: 0.611–0.931) (Table 3).

**Figure 3:**
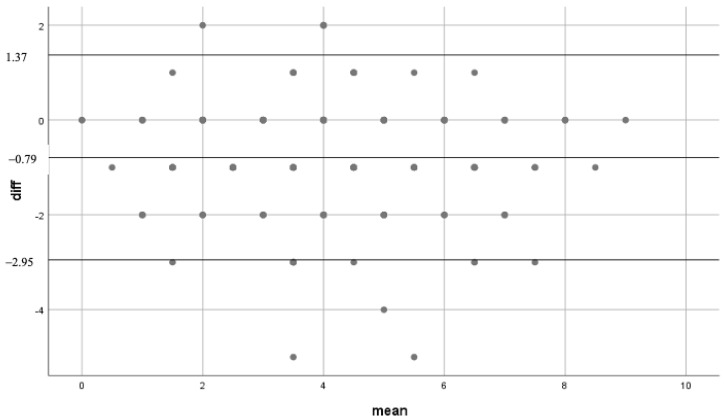
Bland-Altman scatter plot. Mean: mean of the different outcomes of CHA_2_DS_2_-VASc score either aCDSS or mCDSS. Diff: absolute difference in 1 patient between CCDSS and manually calculation.

Bland-Altman analysis indicated a bias of −0.79 with limit of agreement (95%-CI) between −2.95 and 1.37 between the two interventions. Linear regression analysis indicated no significant relation (R = 0.477 (95%-CI: 0.435–0.519), *p* > 0.05) (Figure 3).

**Figure 4:**
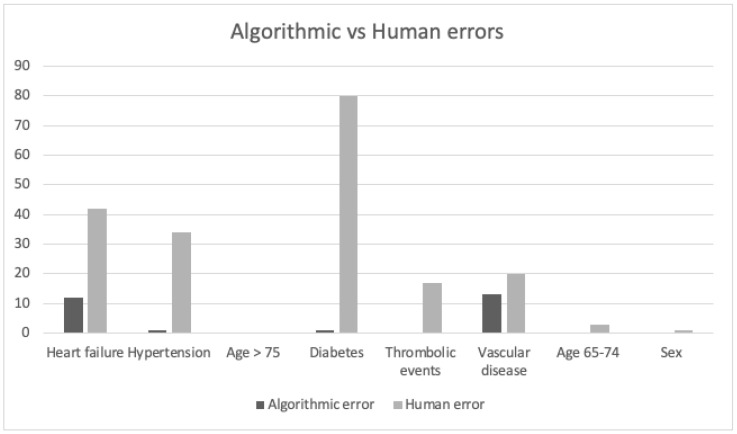
Algorithmic vs. Human errors. Algorithmic error: items aCDSS missed. Human error: items physicians missed (mCDSS). Heart failure: 12 algorithmic errors, 42 human errors. Hypertension: 1 algorithmic error, 34 human errors. Diabetes: 1 algorithmic error, 80 human errors. Thrombo-embolic events: 0 algorithmic errors, 17 human errors. Vascular disease: 13 algorithmic errors, 20 human errors. Age 65–74: 0 algorithmic errors, 3 human errors. Sex: 0 algorithmic errors, 1 human error. From in total 224 errors, were 88% human.

The Cohen’s kappa score was 0.42 for the total CHA_2_DS_2_-VASc score which indicates a moderate level of agreement between the two interventions (Table 1 and Table 2). This result derives from the low correlation between the two interventions for the CHA_2_DS_2_-VASc score criteria: hypertension, diabetes and heart failure (Table 3). A good correlation was found for the CHA_2_DS_2_-VASc score criteria: thromboembolic disorders, symptomatic arteriosclerosis in the legs, and symptomatic coronary disease. A retrospective evaluation performed by the investigators showed that 28% of the calculations had a 100% agreement, 72% did not. Figure 4 shows that mCDSS missed mainly items in heart failure, hypertension and diabetes compared to aCDSS.

The aCDSS took 40 min to calculate all CHA_2_DS_2_-VASc scores of the 224 patients. This is on average 11 s per patient. The three physicians took 180 min to calculate these scores with an average of 48 s per patient. The aCDSS calculated the total CHA_2_DS_2_-VASc score 4.5 times faster compared to physicians.

## 4. Discussion

This study shows a moderate agreement between CHA_2_DS_2_-VASc scores derived mCDSS and aCDSS. The differences between the scores were in 88% of the patients a result of a human error. Besides being more accurate was the calculation with the aCDSS also more time efficient. These findings imply that the application of such an aCDSS can support clinicians during preoperative screening outpatient clinic visit.

Our results are in line with previous studies, which showed that decision support systems can help decrease decision conflicts and increase knowledge of patients with atrial rhythm disturbances about risks atrial rhythm disturbances and treatment options [9,10,11,12,13,14,15]. Others provided evidence that it also helps to improve adherence to guideline in the treatment of atrial fibrillation [11]. In the study of Wang et al. [13] and Silbernagel et al. [13] it was shown that physicians prescribed more anticoagulants due to the clinical decision support system. Moreover, incidence of major bleeding tended to be lower when clinical support systems where used [9]. In our study we also conclude that our aCDSS helps the physician make an accurate decision according to the guideline regarding the use of anticoagulants in perioperative care for patients with atrial fibrillation.

Our results show a moderate agreement between the aCDSS and mCDSS calculated CHA_2_DS_2_-VASc score due to human errors which is a result of the low level of agreement for several criteria of the CHA_2_DS_2_-VASc score. Further analysis showed that the aCDSS included the correct data on different items of the CHA_2_DS_2_-VASc score, whereas during mCDSS, items such as diabetes and heart failure were sometimes overlooked. Therefore, the aCDSS seems to be a more accurate method for calculating the CHA_2_DS_2_-VASc score than mCDSS of the patient health records, while saving time for the clinician.

This study has various limitations. First, this was a retrospective analysis while manual calculations can be performed with the help of a patient during preoperative out-patient clinic visits. Another limitation is that three different physicians divided the 224 patients for mCDSS. Differences of the performance between these physicians might have influenced the results. However, differences between clinicians are common in practice and the constant method of calculations favors the aCDSS. In addition, it will be easier to change practice with the aCDSS if the CHA_2_DS_2_-VASc score change in the future, because with the aCDSS the algorithm can be updated easily while human training takes more time and effort. Bias might have occurred since the physicians were not blinded for the purpose of this study. They may have paid extra attention to retrieve all criteria for calculating the CHA_2_DS_2_-VASc score which may not reflect real practice. The level of agreement might therefore be even smaller. Finally, we do not know whether it is possible that the agreement between the two interventions on individual items in the CHA_2_DS_2_-VASc score is based on chance, but our results after retrospective analysis indicate otherwise.

For the future, an automated calculation of the HAS-BLED score might be added to the algorithm to provide the clinicians a well-balanced advice. Furthermore, a clinical study is needed that evaluates the accuracy, the patient’s and clinician’s benefits, and the usability of a real-time automated CHA_2_DS_2_-VASc score calculations.

## 5. Conclusions

The agreement of a mCDSS CHA_2_DS_2_-VASc score as current standard of care and calculation by an aCDSS is low. This result implies that calculations performed by an aCDSS might be more accurate and more time efficient than a manual calculation. Implementation of this aCDSS in clinical practice can therefore support physicians during their pre-operative outpatient clinics.

## Figures and Tables

**Table 1:** CHA_2_DS_2_-VASc aCDSS/mCDSS cross tabulation. Range CHA_2_DS_2_-VASc. score 0–10. Groups: 0 (no anticoagulants necessary), 1–7 (coagulants necessary, but no need to bridge when temporarily stopped) and >7 (coagulants necessary, but indication to bridge with LMWH (low molecular weight heparins) when temporarily stopped).

		CHA_2_DS_2_-VASc Score mCDSS			
		0	1–7	>7	Total
**CHA_2_DS_2_-VASc score aCDSS**	0	2	0	0	2
	1–7	6	200	0	206
	>7	0	11	5	16
	Total	8	211	5	224

Observer agreement = 207 (92.4%), disagreement = 7.6%.

**Table 2:** Calculation for Cohen’s kappa based on Table 2. Range CHA_2_DS_2_-VASc score 0–10.

CHA_2_DS_2_-VASc score	0	1–7	>7
Chance agreement	0.071	194.045	0.357

Observed agreement = 92.4%. Chance agreement between both methods = (0.071 + 194.045 + 0.357)/224 = 0.868 (86.8%). For chance corrected observation agreement = 92.4 − 86.8 = 5.6%. For chance corrected potential agreement = 100 − 86.8% = 13.2%. Kappa = 5.6%/13.2% = 0.424.

**Table 3:** Interclass Correlation Coefficient (95%-CI); aCDSS vs mCDSS CHA_2_DS_2_-VASc score.

Item CHA_2_DS_2_-VASc Score	Type Measurement	Interclass Correlation Coefficient
Total CHA_2_DS_2_-VASc score	Single	0.754 (0.440–0.871)
	Average	0.859 (0.661–0.931)
Hypertension	Single	0.531 (0.387–0.642)
	Average	0.693 (0.558–0.782)
Diabetes	Single	0.305 (0.056–0.495)
	Average	0.467 (0.106–0.662)
Thromboembolic events	Single	0.769 (0.705–0.820)
	Average	0.870 (0.827–0.901)
Heart failure	Single	0.396 (0.274–0.505)
	Average	0.567 (0.430–0.671)
symptomatic arteriosclerosis in the legs and symptomatic coronary disease	Single	0.705 (0.633–0.766)
	Average	0.827 (0.775–0.867)

More details are shown in Supplementary S2 (Tables S1–S6). Range of CHA_2_DS_2_-VASc score is 0–10.

## Data Availability

Not applicable.

## Supplementary Materials

The following supporting information can be downloaded at: https://www.mdpi.com/article/10.3390/medicina58091269/s1, Supplementary S1: Search items in EZIS; Supplementary S2: Extra tables.
