# Supplementary material for: Quality Improvement in the Preoperative Evaluation: Accuracy of an Automated Clinical Decision Support System to Calculate CHA2DS2-VASc Scores
Source: Medicina (Kaunas). 2022 Sep 13;58(9):1269. doi: 10.3390/medicina58091269 (PMC9500878; doi:10.3390/medicina58091269)
Supplement: Supplementary file 1 [file medicina-58-01269-s001.zip › medicina-1852902-supplementary.pdf]

Supplementary S1:

Search items in EZIS (partly in Dutch because of the language of the doctor))

Hypertension: definition hypertension was determined by: Am J Epidemiol. 1989 Apr;129(4):687-702. The Atherosclerosis Risk in Communities (ARIC) Study: design and objectives. The ARIC investigators.

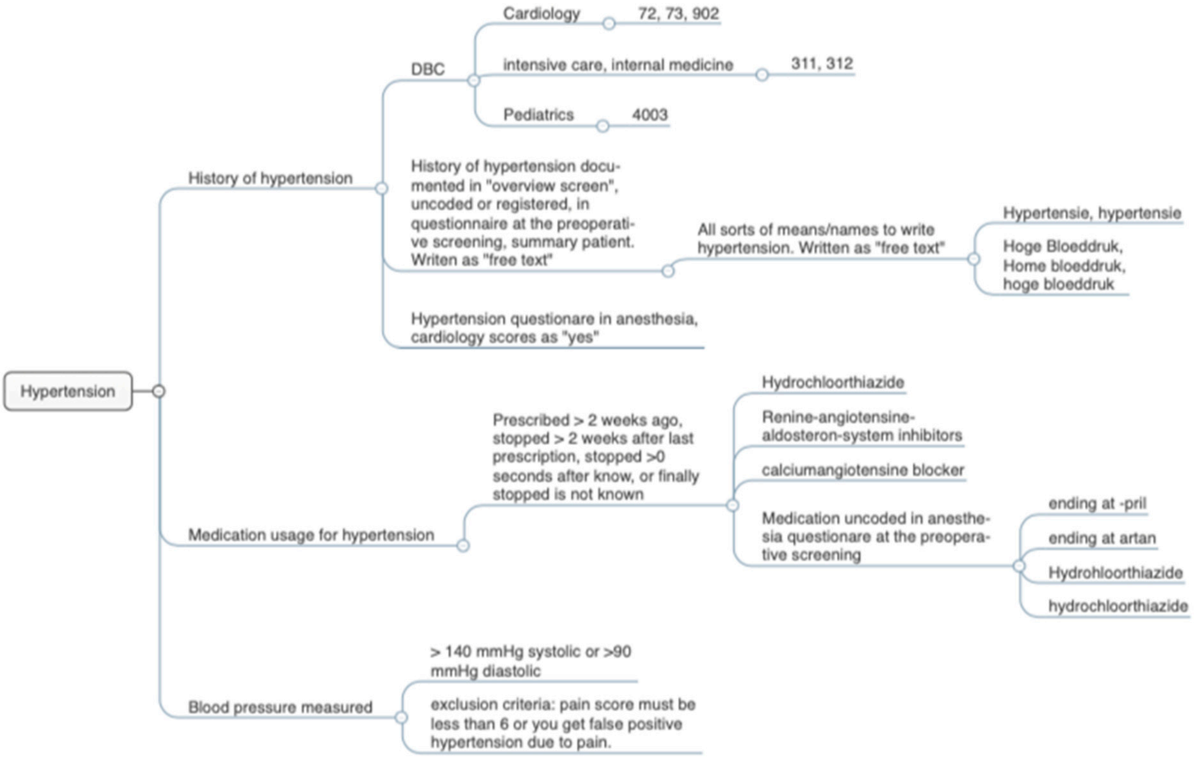

Diabetes:

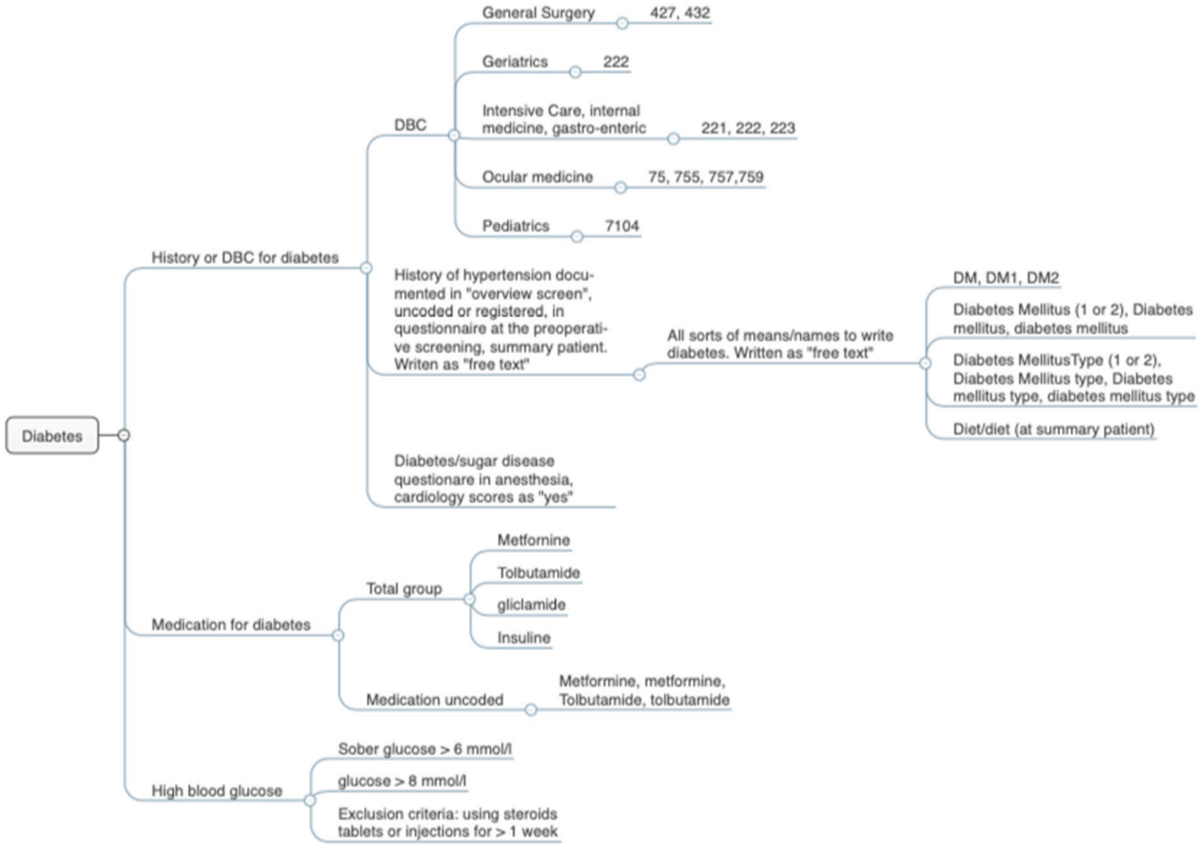
Diabetes
History or DBC for diabetes
DBC
General Surgery
427, 432
Geriatrics
222
Intensive Care, internal medicine, gastro-enteric
221, 222, 223
Ocular medicine
75, 755, 757,759
Pediatrics
7104
History of hypertension documented in "overview screen", uncoded or registered, in questionnaire at the preoperative screening, summary patient. Writen as "free text"
All sorts of means/names to write diabetes. Written as "free text"
DM, DM1, DM2
Diabetes Mellitus (1 or 2), Diabetes mellitus, diabetes mellitus
Diabetes MellitusType (1 or 2), Diabetes Mellitus type, Diabetes mellitus type, diabetes mellitus type
Diet/diet (at summary patient)
Diabetes/sugar disease questionare in anesthesia, cardiology scores as "yes"
Medication for diabetes
Total group
Metformine
Tolbutamide
gliclamide
Insuline
Medication uncoded
Metformine, metformine, Tolbutamide, tolbutamide
High blood glucose
Sober glucose > 6 mmol/l
glucose > 8 mmol/l
Exclusion criteria: using steroids tablets or injections for > 1 week

Thromboembolic disorders:

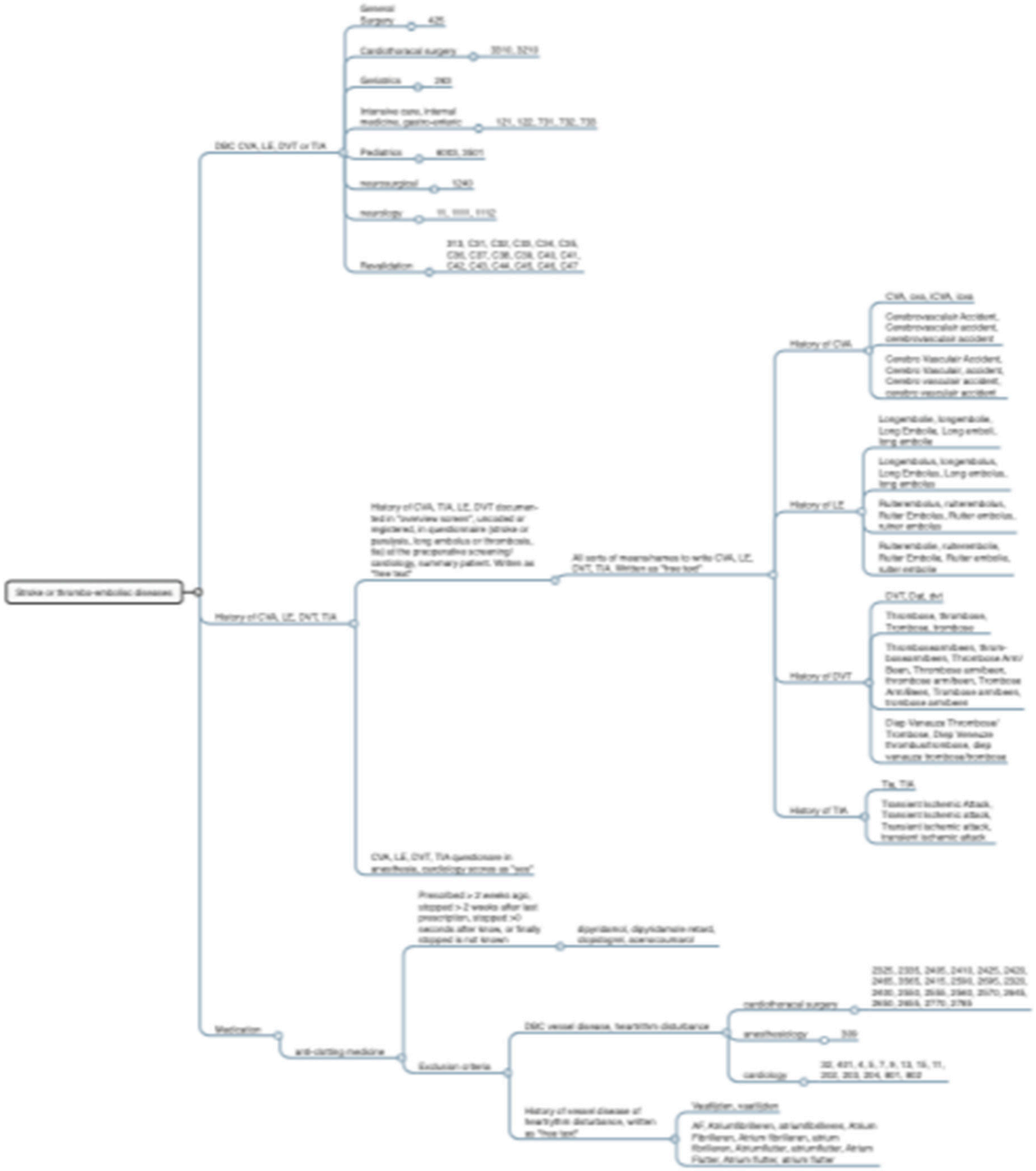

Zomed in:

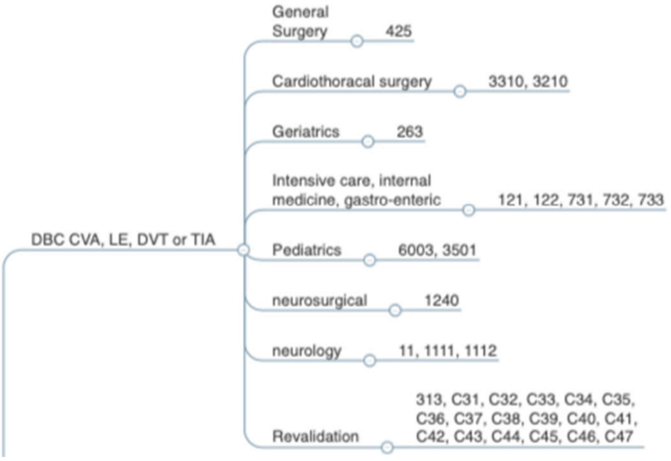

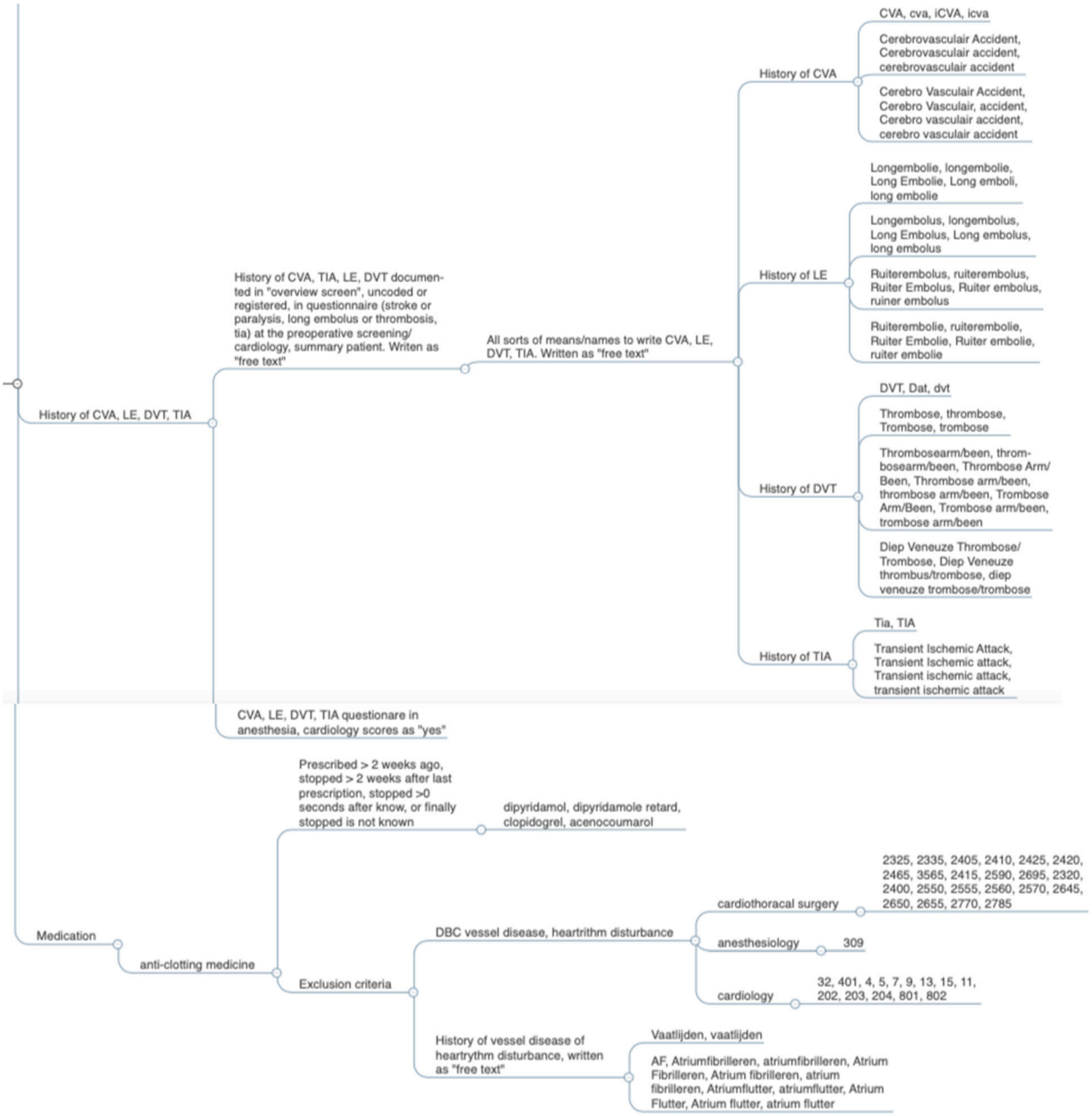
History of CVA, LE, DVT, TIA
History of CVA, TIA, LE, DVT documented in "overview screen", uncoded or registered, in questionnaire (stroke or paralysis, long embolus or thrombosis, tia) at the preoperative screening/ cardiology, summary patient. Writen as "free text"
All sorts of means/names to write CVA, LE, DVT, TIA. Written as "free text"
History of CVA
CVA, cva, iCVA, icva
Cerebrovasculair Accident, Cerebrovasculair accident, cerebrovasculair accident
Cerebro Vasculair Accident, Cerebro Vasculair, accident, Cerebro vasculair accident, cerebro vasculair accident
History of LE
Longembolie, longembolie, Long Embolie, Long emboli, long embolie
Longembolus, longembolus, Long Embolus, Long embolus, long embolus
Ruiterembolus, ruiterembolus, Ruiter Embolus, Ruiter embolus, ruiner embolus
Ruiterembolie, ruiterembolie, Ruiter Embolie, Ruiter embolie, ruiter embolie
History of DVT
DVT, Dat, dvt
Thrombose, thrombose, Trombose, trombose
Thrombosearm/been, thrombosearm/been, Thrombose Arm/ Been, Thrombose arm/been, thrombose arm/been, Trombose Arm/Been, Trombose arm/been, trombose arm/been
Diep Veneuze Thrombose/ Trombose, Diep Veneuze thrombus/trombose, diep veneuze trombose/trombose
History of TIA
Tia, TIA
Transient Ischemic Attack, Transient Ischemic attack, Transient ischemic attack, transient ischemic attack
CVA, LE, DVT, TIA questionare in anesthesia, cardiology scores as "yes"
Medication
anti-clotting medicine
Prescribed > 2 weeks ago, stopped > 2 weeks after last prescription, stopped >0 seconds after know, or finally stopped is not known
dipyridamol, dipyridamole retard, clopidogrel, acenocoumarol
Exclusion criteria
DBC vessel disease, heartrithm disturbance
cardiothoracal surgery
2325, 2335, 2405, 2410, 2425, 2420, 2465, 3565, 2415, 2590, 2695, 2320, 2400, 2550, 2555, 2560, 2570, 2645, 2650, 2655, 2770, 2785
anesthesiology
309
cardiology
32, 401, 4, 5, 7, 9, 13, 15, 11, 202, 203, 204, 801, 802
History of vessel disease of heartrythm disturbance, written as "free text"
Vaatlijden, vaatlijden
AF, Atriumfibrilleren, atriumfibrilleren, Atrium Fibrilleren, Atrium fibrilleren, atrium fibrilleren, Atriumflutter, atriumflutter, Atrium Flutter, Atrium flutter, atrium flutter

Heart failure:

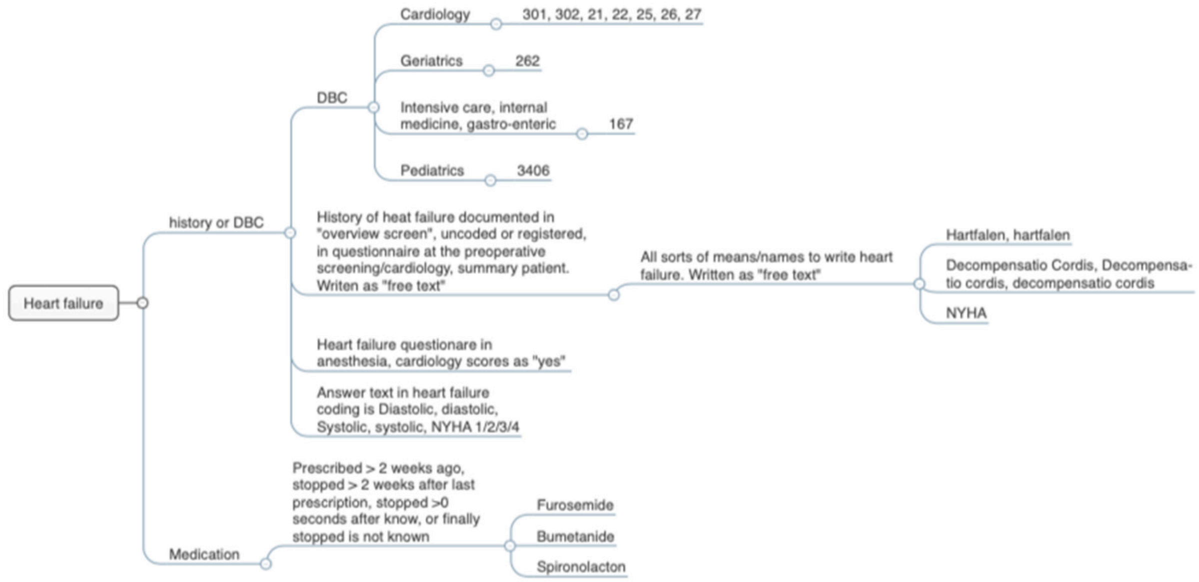

Symptomatic arteriosclerosis in the legs and symptomatic coronary disease:

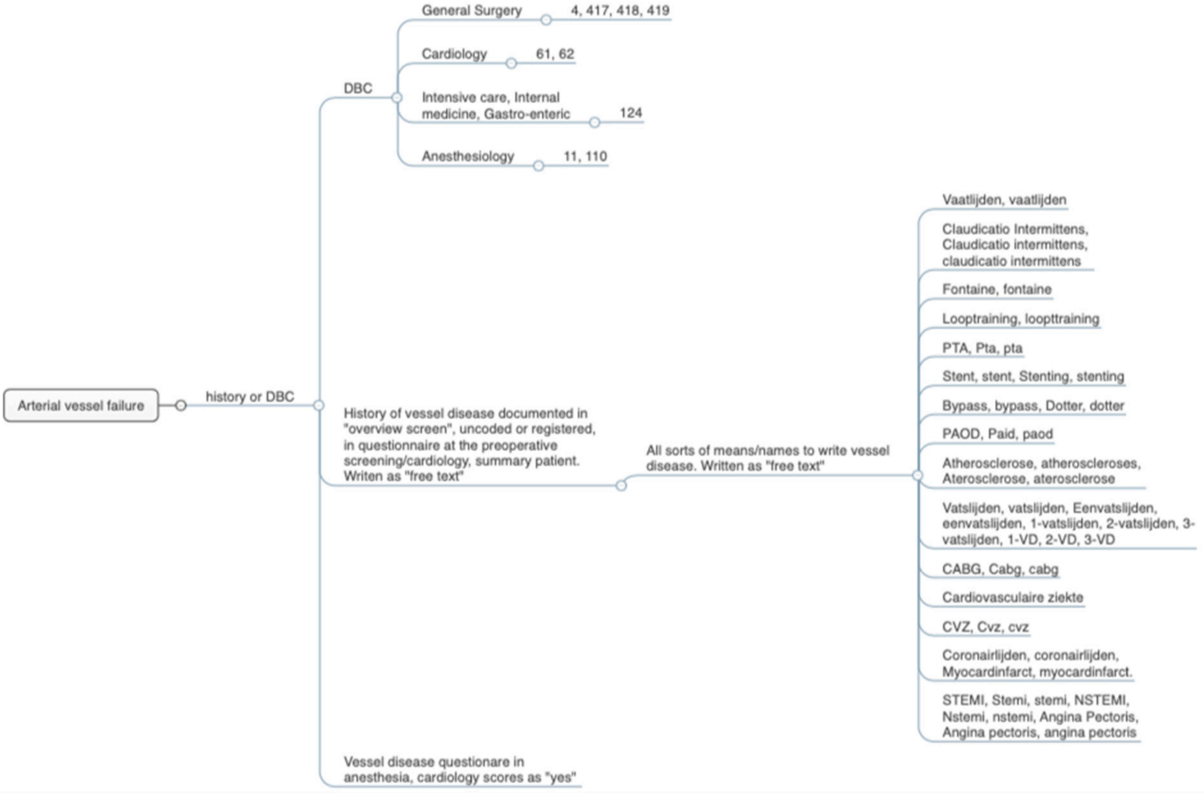

Supplementary S2:

Table S1: interclass correlation coefficient for aCDSS vs mCDSS calculation for total CHA_2DS_2-VASc score

Intraclass Correlation Coefficient

	Intraclass Correlation[b]	95% Confidence Interval		F Test with True Value 0			
		Lower Bound	Upper Bound	Value	df1	df2	Sig
Single Measures	,754[a]	,440	,871	10,231	223	223	,000
Average Measures	,859[c]	,611	,931	10,231	223	223	,000

Two-way mixed effects model where people effects are random and measures effects are fixed.

a. The estimator is the same, whether the interaction effect is present or not.

b. Type A intraclass correlation coefficients using an absolute agreement definition.

c. This estimate is computed assuming the interaction effect is absent, because it is not estimable otherwise.

Table S2: aCDSS vs mCDSS calculation hypertension

Intraclass Correlation Coefficient

	Intraclass Correlation[b]	95% Confidence Interval		F Test with True Value 0			
		Lower Bound	Upper Bound	Value	df1	df2	Sig
Single Measures	,531[a]	,387	,642	3,601	223	223	,000
Average Measures	,693[c]	,558	,782	3,601	223	223	,000

Two-way mixed effects model where people effects are random and measures effects are fixed.

a. The estimator is the same, whether the interaction effect is present or not.

b. Type A intraclass correlation coefficients using an absolute agreement definition.

c. This estimate is computed assuming the interaction effect is absent, because it is not estimable otherwise.

Table S3: aCDSS vs mCDSS calculation diabetes

Intraclass Correlation Coefficient

	Intraclass Correlation[b]	95% Confidence Interval		F Test with True Value 0			
		Lower Bound	Upper Bound	Value	df1	df2	Sig
Single Measures	,305[a]	,056	,495	2,333	223	223	,000
Average Measures	,467[c]	,106	,662	2,333	223	223	,000

Two-way mixed effects model where people effects are random and measures effects are fixed.

a. The estimator is the same, whether the interaction effect is present or not.

b. Type A intraclass correlation coefficients using an absolute agreement definition.

c. This estimate is computed assuming the interaction effect is absent, because it is not estimable otherwise.

Table S4: aCDSS vs mCDSS calculation thromboembolic events

Intraclass Correlation Coefficient

	Intraclass Correlation[b]	95% Confidence Interval		F Test with True Value 0			
		Lower Bound	Upper Bound	Value	df1	df2	Sig
Single Measures	,769[a]	,705	,820	7,953	223	223	,000
Average Measures	,870[c]	,827	,901	7,953	223	223	,000

Two-way mixed effects model where people effects are random and measures effects are fixed.

a. The estimator is the same, whether the interaction effect is present or not.

b. Type A intraclass correlation coefficients using an absolute agreement definition.

c. This estimate is computed assuming the interaction effect is absent, because it is not estimable otherwise.

Table S5: aCDSS vs mCDSS calculation heart failure

Intraclass Correlation Coefficient

	Intraclass Correlation[b]	95% Confidence Interval		F Test with True Value 0			
		Lower Bound	Upper Bound	Value	df1	df2	Sig
Single Measures	,396[a]	,274	,505	2,407	223	223	,000
Average Measures	,567[c]	,430	,671	2,407	223	223	,000

Two-way mixed effects model where people effects are random and measures effects are fixed.

a. The estimator is the same, whether the interaction effect is present or not.

b. Type A intraclass correlation coefficients using an absolute agreement definition.

c. This estimate is computed assuming the interaction effect is absent, because it is not estimable otherwise.

Table S6: aCDSS vs mCDSS calculation symptomatic arteriosclerosis in the legs and symptomatic coronary disease

Intraclass Correlation Coefficient

	Intraclass Correlation[b]	95% Confidence Interval		F Test with True Value 0			
		Lower Bound	Upper Bound	Value	df1	df2	Sig
Single Measures	,705[a]	,633	,766	5,821	223	223	,000
Average Measures	,827[c]	,775	,867	5,821	223	223	,000

Two-way mixed effects model where people effects are random and measures effects are fixed.

a. The estimator is the same, whether the interaction effect is present or not.

b. Type A intraclass correlation coefficients using an absolute agreement definition.

c. This estimate is computed assuming the interaction effect is absent, because it is not estimable otherwise.

Table S7: one-sample test of the difference between aCDSS vs mCDSS.

One-Sample Statistics

	N	Mean	Std. Deviation	Std. Error Mean
diff	224	-,79	1,103	,074

One-Sample Test

	Test Value = 0					
					95% Confidence Interval of the Difference	
	t	df	Sig. (2-tailed)	Mean Difference	Lower	Upper
diff	-10,725	223	,000	-,790	-,94	-,64

Table S8: Regression analysis of the Bland-Altman scatter plot.

Coefficients[a]

Model		Unstandardized Coefficients		Standardized Coefficients	t	Sig.
		B	Std. Error	Beta		
1	(Constant)	-,668	,187		-3,570	,000
	mean	-,030	,042	-,048	-,713	,477

a. Dependent Variable: diff
